# The novel missense mutation Met48Lys in FKBP22 changes its structure and functions

**DOI:** 10.1038/s41598-019-57374-y

**Published:** 2020-01-16

**Authors:** Yoshihiro Ishikawa, Nobuyo Mizuno, Paul Holden, Pei Jin Lim, Douglas B. Gould, Marianne Rohrbach, Cecilia Giunta, Hans Peter Bächinger

**Affiliations:** 1 Department of Biochemistry and Molecular Biology, Oregon Health & Science University, Portland, OR USA; 2 Shriners Hospital for Children, Research Department, Portland, OR USA; 3 Department of Ophthalmology, University of California, School of Medicine, San Francisco, CA USA; 4 Vaccine and Gene Therapy Institute, Oregon Health and Science University, Portland, Oregon USA; 5 Connective Tissue Unit, Division of Metabolism and Children’s Research Centre, University Children’s Hospital, Zürich, Switzerland; 6 Department of Anatomy, University of California, School of Medicine, San Francisco, CA USA

**Keywords:** Endoplasmic reticulum, Endoplasmic reticulum, Endoplasmic reticulum, Endoplasmic reticulum

## Abstract

Mutations in the *FKBP14* gene encoding FKBP22 (FK506 Binding Protein 22 kDa) cause kyphoscoliotic Ehlers-Danlos Syndrome (kEDS). The first clinical report showed that a lack of FKBP22 protein due to mutations causing nonsense-mediated decay of the mRNA leads to a wide spectrum of clinical phenotypes including progressive kyphoscoliosis, joint hypermobility, hypotonia, hyperelastic skin, hearing loss and aortic rupture. Our previous work showed that these phenotypic features could be correlated with the functions of FKBP22, which preferentially binds to type III, VI and X collagens, but not to type I, II or V collagens. We also showed that FKBP22 catalyzed the folding of type III collagen through its prolyl isomerase activity and acted as a molecular chaperone for type III collagen. Recently, a novel missense mutation Met48Lys in FKBP22 was identified in a patient with kEDS. In this report, we expand the list of substrates of FKBP22 and also demonstrate that the Met48Lys mutation diminishes the activities of FKBP22, indicating that pathology can arise from absence of FKBP22, or partial loss of its function.

## Introduction

Ehlers-Danlos syndrome (EDS) is an inherited connective tissue disorder mainly characterized by joint hypermobility, skin hyperextensibility, and tissue fragility. Thirteen subtypes were classified in the 2017 EDS Nosology^1^ and the kyphoscoliotic EDS (kEDS) was recognized as one subtype demonstrating a broader clinical spectrum of phenotypes with major and minor defined criteria. Congenital hypotonia, congenital or early onset kyphoscoliosis, and generalized joint hypermobility with dislocations/subluxations are the major features, and the minor features include skin hyperextensibility, easily bruised skin, rupture/aneurysm of a medium-sized arteries, and osteopenia/osteoporosis among others^1^. While the musculoskeletal symptoms are found in disorders caused by mutations in extracellular matrix (ECM) proteins, in particular collagen which is the most abundant protein in humans^2,3^, kEDS results from the deficiency of two proteins participating in collagen biosynthesis in the rough Endoplasmic Reticulum (rER): the post-translational modifying enzyme Lysyl Hydroxylase 1 (LH1 encoded by *PLOD1*)^4,5^ and the peptidyl-prolyl cis/trans isomerase (PPIase) family protein FK506-binding protein 22 kDa (FKBP22 encoded by *FKBP14*)^6,7^. The lack of these proteins changes the collagen biosynthetic molecular ensemble in the rER^8–10^, which can lead to the secretion of structurally and/or functionally abnormal collagens^11,12^. Therefore, EDS causative mutations are not restricted to genes coding for ECM molecules but also include genes coding for proteins required for ECM biosynthesis and processing.

Proteins are biosynthesized inside the cells following the central dogma that protein information stored in DNA is first transcribed to messenger RNA (mRNA) which is then translated into protein^13,14^. Missense mutations affect this central dogma and alter protein biogenesis in different ways. Depending upon the location of the mutation, protein expression can be completely lost due to nonsense-mediated mRNA decay, or a missense mutation producing a polypeptide chain containing an incorrect amino acid sequence may occur^15,16^. To date, over twenty kEDS cases with *FKBP14* mutation have been reported and all of these mutations lead to a complete loss of FKBP22 through nonsense-mediated mRNA decay. More recently, a patient was identified with a novel homozygous c.143 T > A substitution in exon 1 of *FKBP14*^6,7^, that is predicted to produce an FKBP22 protein with a Met48Lys (M48K).

FKBP22 is a rER-resident PPIase that preferentially catalyzes the posttranslational modification of polypeptide chains containing hydroxyproline rather than proline residues and accelerates the rate of collagen folding *in vitro*^17,18^. Moreover, FKBP22 shows collagen type-specific interactions suggesting a potential role as a molecular chaperone, which helps to explain the broader phenotypic features of kEDS described above^18^. However, little is known about which particular function of FKBP22 is critical to cause kEDS.

Here, we aimed to investigate the functional consequence of the M48K missense mutation in FKBP22. We performed biochemical characterizations of recombinant purified M48K and wild type (WT) FKBP22 proteins and compared the intracellular localization and solubility of mutant and WT proteins in the cells. Our results indicate that the mutant M48K FKBP22 is stable and localized to the rER, but the functions are clearly impaired. We suggest that the M48K mutation alters the local environment of the FKBP22 structure and impairs the protein functions.

## Results

### Structural comparison using a homology model

**Figure 1:**
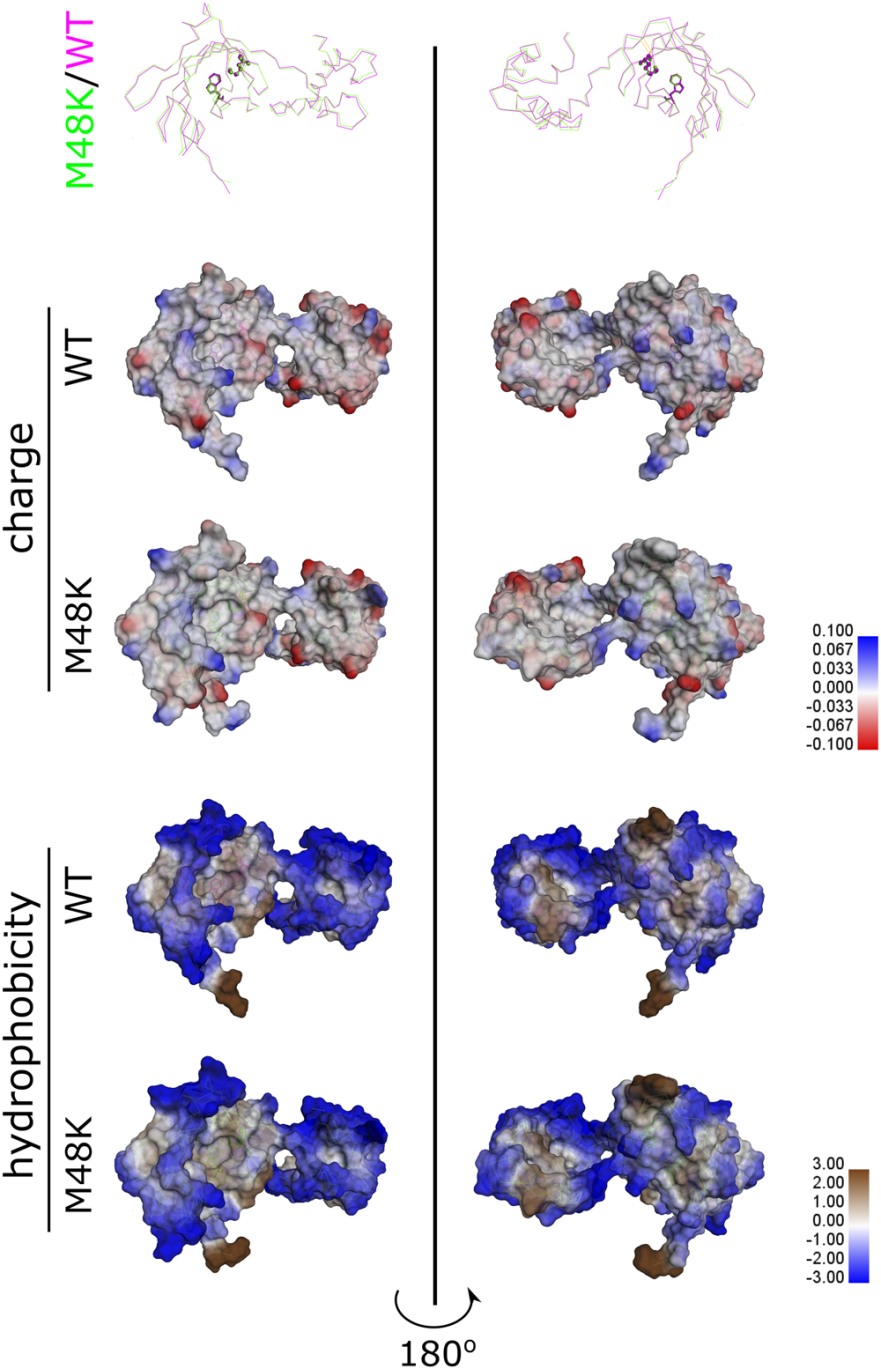
Structural comparison between WT and M48K FKBP22. The structural homology model of M48K FKBP22 was created using the known WT FKBP22 structure (PDB: 4MSP). Top – The overall structures of WT and M48K FKBP22 are aligned using a Cα wire style. The atoms affected by the mutation (Met48 and Lys48) are shown in ball and stick, and the tryptophan residue (W88) which is the located at the bottom of the active site of FKBP domain is displayed as a stick. Middle – The surface charge states are shown for both WT and M48K FKBP22. The surface colors indicate the interpolated atomic charge of the receptor atoms, from blue for positive to red for negative. Bottom – The surface hydrophobicity is shown for both WT and M48K FKBP22. The surface colors indicate the hydrophobicity of the receptor residues, from blue for hydrophilic to brown for hydrophobic.

We developed a structural model of M48K FKBP22 using the WT crystal structure previously reported^19^ to see if the structure of the mutant protein is different to WT. The overall structure of WT and M48K FKBP22 are similar and although the mutation is close to the active site of the FKBP domain, it does not significantly change the spatial arrangement of the FKBP domain (top in Fig. 1). However, the surface charge and hydrophobicity are slightly altered by the M48K substitution (middle and bottom in Fig. 1), thus suggesting that the microenvironment around the active site may be changed by the mutation. We hypothesize that M48K FKBP22 should fold normally in the rER but that its function may be impaired by subtle structural change at the active site.

### Basic characterization of recombinant human M48K FKBP22

**Figure 2:**
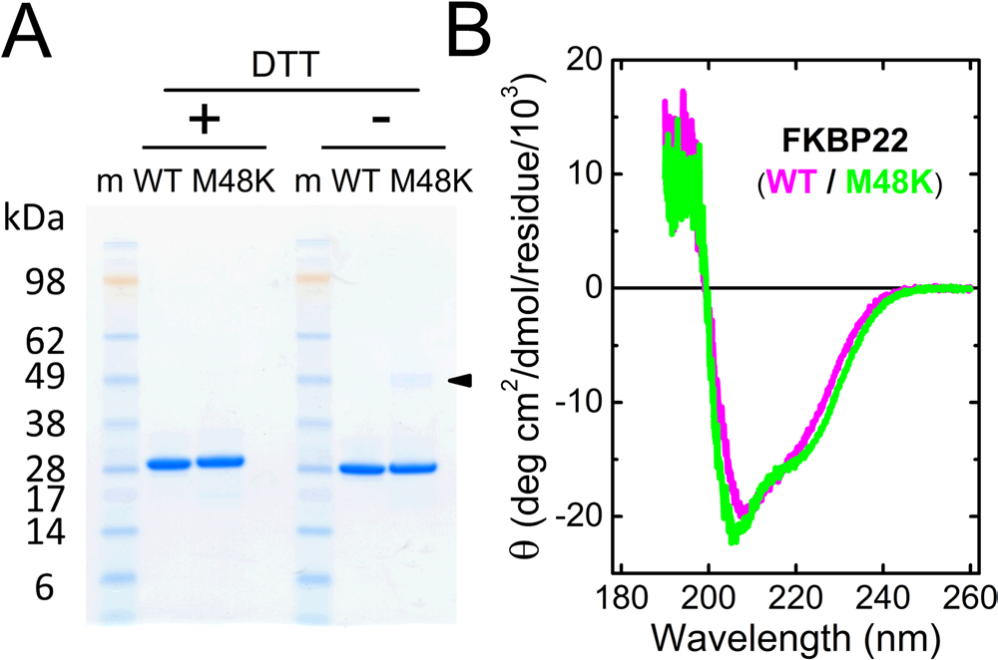
Characterization of recombinant human WT and M48K FKBP22. (**A**) SDS/PAGE analysis of purified recombinant human WT and M48K FKBP22. The recombinant proteins were purified from an *E. coli* expression system, and the figure shows the final purified material in the presence (+) and absence (−) of DTT running on a Bolt 4–12% Bis-Tris plus gel (Thermo Fisher Scientific) stained with GelCode Blue Stain Reagent (Thermo Fisher Scientific). Arrowhead points to the small aggregates formed by mutant FKBP22. The image of SDS/PAGE gel was scanned by EPSON Perfection V700 photo and then the original scanned image was used to create this figure. (**B**) CD spectra of human WT (Magenta) and M48K (Green) FKBP22. The CD spectra were measured at 4 °C in 1 mM Tris buffer, containing 0.05 mM CaCl_2_, pH 7.5.

To characterize the effect of the M48K mutation on FKBP22 function, we introduced the mutation into an existing WT FKBP22 construct for expression in *E. coli*^18^. Figure 2A shows an SDS-polyacrylamide gel of purified recombinant human WT and M48K FKBP22 proteins with and without reducing agent dithiothreitol (DTT). Both proteins were isolated to a high level of purity and showed similar migration on the gel. However, urea was needed to help solubilize the mutant but not WT FKBP22 when it was extracted from *E. coli* cell pellets, despite the similar yield of protein expression in both WT and M48K FKBP22. This indicates that the mutant FKBP22 protein tends to form aggregates more readily than WT FKBP22. Therefore, a very small amount of mutant FKBP22 could form a dimer or aggregates under non-reducing conditions even in the final purified form (Arrowhead in Fig. 2). For a structural comparison, circular dichroism (CD) spectra were measured (Fig. 2B). Small differences were observed in their CD spectra at around 200–240 nm, however the overall secondary structures looked very similar in agreement with the homology model we showed in Fig. 1.

### Functional assessment of recombinant human WT and M48K FKBP22

**Figure 3:**
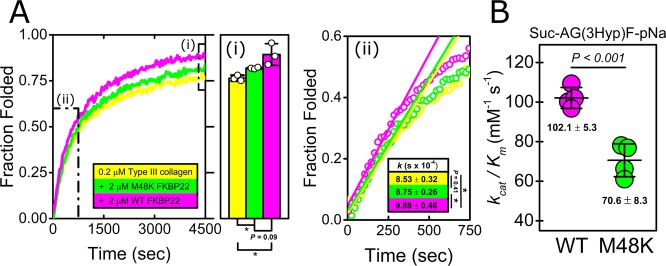
Comparison of catalytic efficiency for the prolyl isomerization between WT and M48K FKBP22. (**A**) Kinetics of the refolding of type III collagen in the presence of recombinant human WT and M48K FKBP22 monitored by circular dichroism at 220 nm is shown. All curves are averaged from a minimum of three measurements. Area (i) in the CD plot highlights the amount of refolded type III collagen at the end of measurements, also shown as a bar chart to the right. Area (ii) indicates the initial folding rate of type III collagen, also shown in more detail to the far right. Open circles and solid straight lines represent raw data points and calculated initial folding rate, respectively. The slope of the straight lines reflects the initial rate of folding of type III collagen. (**B**) Catalytic efficiency for the peptidyl-prolyl isomerization of a peptide substrate by recombinant human WT (Magenta) and M48K (Green) FKBP22. The catalytic efficiency (*k*_*cat*_/*K*_*m*_: mM^−1^·s^−1^) for the isomerization reaction was determined by kinetic measurements using the Suc-Ala-Gly-3Hyp-Phe-pNa peptide substrate, which showed the highest efficiency in previous experiments. The numbers in the graph indicate the mean ± S.D. and the *P* value (*P* < 0.05) is annotated as an asterisk in area (i) and (ii).

To investigate the effect of the mutation on FKBP22 functions, we performed biochemical assays using the characterized recombinant proteins shown in Fig. 2. Two major functions have previously been determined for WT FKBP22 during collagen biosynthesis in the rER: PPIase activity and collagen binding ability^18^. Therefore, we first examined *in vitro* collagen refolding in the presence and absence of WT and M48K FKBP22 since collagen folding is accelerated by PPIase activities^10,20^. Experiments were performed using CD with type III collagen as a substrate as described previously^17,21^. A higher amount of final folded product was seen in the presence of WT FKBP22 (magenta, Fig. 3A) and mutant M48K FKBP22 (green, Fig. 3A) compared to control without FKBP22 (yellow, Fig. 3A), however the M48K mutant protein was less efficient than with WT. A significantly faster rate of refolding was also observed in the presence of WT FKBP22, while that of M48K FKBP22 was only marginally higher than control. Therefore, the mutation appears to reduce, but not abolish, the PPIase activity of FKBP22. We thus decided to quantify the level of PPIase activity of M48K FKBP22 relative to that of WT FKBP22. We previously quantitated the level of PPIase activities of six rER resident PPIases using proline or hydroxyproline containing peptide substrates *in vitro*^17^. In these studies, FKBP22 showed a greater catalytic efficiency towards hydroxyproline containing substrates than to proline containing substrates and the highest PPIase activity was detected towards a peptide containing glycine and 3-hydroxyproline^17^. Therefore, we used this same glycine and 3-hydroxyproline containing peptide (Suc-Ala-Gly-3Hyp-Phe-pNa) to compare the catalytic efficiency between WT and M48K FKBP22. As shown in Fig. 3B, the mutation clearly leads to a decrease in catalytic efficiency of approximately 30% when compared to WT.

**Figure 4:**
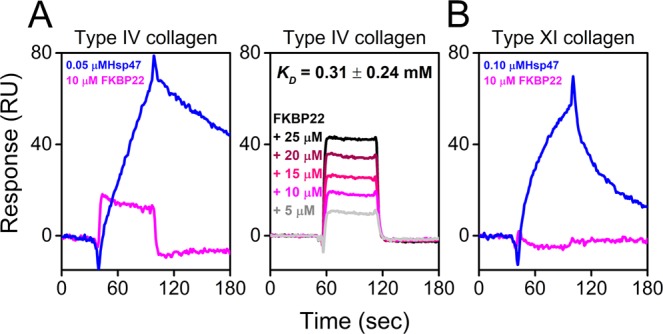
Direct binding kinetics of WT human FKBP22 to collagens. Direct binding kinetics were measured by SPR analysis using a BIAcore X instrument. Human WT FKBP22 was injected over CM5 chips on which (**A**) mouse type IV or (**B**) bovine type XI collagen had been immobilized. Hsp47 was used as a positive control. Titrating concentrations of WT FKBP22 were run over the mouse type IV collagen chip to determine the *K*_*D*_ value.

**Figure 5:**
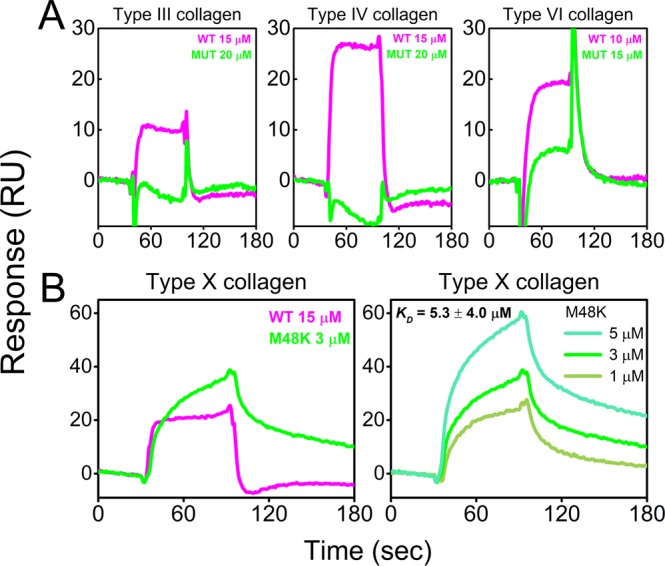
Interactions of collagens with recombinant human WT and M48K FKBP22. Direct binding kinetics were measured by SPR analysis using a BIAcore X instrument. Collagens, (**A**) bovine type III, mouse type IV and human type VI and (**B**) human type X, which had previously shown positive binding to WT FKBP22, were immobilized on CM5 chips and recombinant human WT and M48K FKBP22 were injected to compare their binding activities. Titrating concentrations of M48K FKBP22 were run over the human type X collagen chip to determine the *K*_*D*_ value.

Next, we performed protein – protein interaction studies to determine if the mutation also affects the collagen binding ability of FKBP22. This was important to study because the binding of FKBP22 to type III, VI, and X collagen but not to types I, II, and V is proposed to account for the broader spectrum of the phenotype in patients with FKBP22 mutations^18^. Since there are still unresolved phenotypes resulting from a lack of FKBP22, we took this opportunity to expand on our previously identified FKBP22 interacting collagen substrates by testing two additional and physiologically relevant collagens. We prepared type IV and type XI collagen which are mainly located in basement membranes^22,23^ and articular cartilage^24,25^, respectively. Surface plasmon resonance (SPR) analysis was performed using type IV and XI collagen immobilized on CM5 chips. The collagen binding molecular chaperone, Hsp47, was used as a positive control. WT FKBP22 clearly interacted with type IV collagen showing a concentration-dependent binding (Fig. 4A), whereas no binding was detected to type XI collagen (Fig. 4B). However, the equilibrium dissociation constant (K_D_) in Fig. 4A showed that the binding of WT FKBP22 to type IV collagen (0.31 ± 0.24 mM) was much weaker than that to the other three collagen types previously reported to interact with FKBP22 (0.069 mM for type III, 0.062 mM for type VI and 0.043 mM for type X collagen)^18^. In total we were able to carry out SPR measurements toward four different collagen types using WT and M48K FKBP22. Figure 5 shows direct binding kinetics of WT and M48K FKBP22 to collagens. In a similar manner as with PPIase activity, M48K FKBP22 still interacted with collagens but the binding was much weaker than WT FKBP22 to type III, IV and VI collagen (Fig. 5A). Surprisingly, the binding of M48K FKBP22 to type X collagen was stronger than that of WT FKBP22 as shown by the very slow dissociation kinetics (Fig. 5B). The calculated K_D_ value of M48K FKBP22 to type X collagen (5.3 ± 4.0 µM) in Fig. 5B is smaller than that of WT FKBP22 (43 µM) previously reported^18^.

In summary, the M48K mutation in FKBP22 has a subtle influence on its structure and weakens both its PPIase activity and collagen binding properties *in vitro*.

### Intracellular localization and solubility of M48K FKBP22

**Figure 6:**
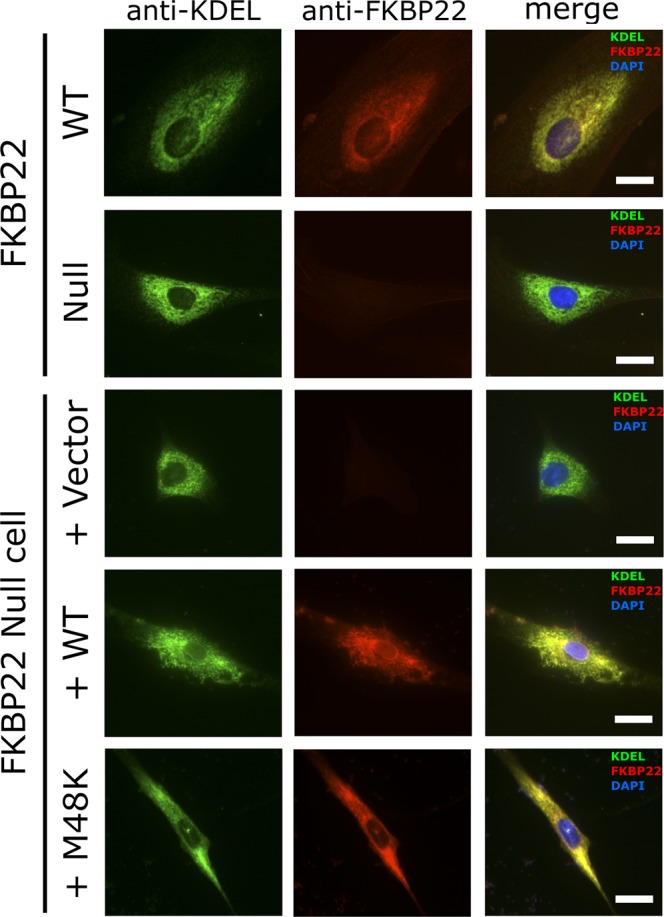
Immunofluorescence staining to determine the subcellular localization of human WT and M48K FKBP22. FKBP22 Null human primary fibroblasts with and without transfection and WT human primary fibroblasts were stained against anti-KDEL (Green) and anti-FKBP22 (Red). Purple color indicates DAPI staining. Scale bars: 20 µm.

**Figure 7:**
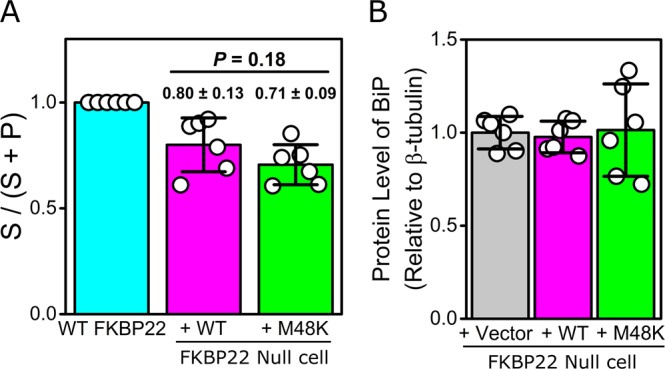
Intracellular solubility of human endogenous WT FKBP22, transfected WT and M48K FKBP22 and protein level of BiP in transfected FKBP22 Null human primary fibroblasts. (**A**) A quantitative analysis of intracellular solubility of human endogenous WT FKBP22, transfected WT and M48K FKBP22 using Western blotting. S and P indicate the supernatant and pellet fraction after centrifugation. The ratio of soluble portion was calculated by total protein amounts combined in S and P. Data presented are means ± SD and individual data points represent independently prepared cell lysates (n = 6). Representative images of western blotting used for this analysis is shown in Supplemental Fig. S1A,B. (**B**). A quantitative analysis of protein level of BiP in transfected FKBP22 Null human primary fibroblasts using Western blotting. The protein signals of BiP were normalized to β-tubulin levels and the signals of transfected vector control were set to 1 to compare with that of the transfected human WT and M48K FKBP22. Data presented are means ± SD and individual data points represent independently prepared cell lysates (n = 6). Representative images of western blotting used for this analysis is shown in Supplemental Fig. S1C.

To confirm that our *in vitro* results could possibly explain the molecular pathology of kEDS caused by M48K mutation in FKBP22, we examined the subcellular localization and intracellular solubility of the M48K FKBP22 in the cells. Since M48K FKBP22 patient cells are unavailable, we transfected human WT and M48K expression constructs in FKBP22 null human fibroblasts, which were established by explant culture from a skin biopsy from a patient with a Glu122ArgfsTer7 (c.362dupC) mutation. The intracellular localization of M48K FKBP22 was determined by immunofluorescent staining of the endogenous or transfected FKBP22 protein co-stained with an antibody specific for the ER retention signal KDEL. Although cell morphology was altered in transfected cells, both transfected WT and M48K FKBP22 properly co-localized with the KDEL containing ER resident proteins as well as endogenous WT FKBP22. (Fig. 6). Next, we evaluated the solubility of M48K FKBP22 in the rER by Western blot analysis. Lysed cells were separated into the supernatant (S) and pellet (P) fractions using centrifugation and we calculated the ratio of soluble portion in total protein amounts combined in S and P. Endogenous FKBP22 in WT cell showed two bands in western blotting (Supplemental Fig. S1A). Since there is a *N*-glycosylation site in FKBP22 (^176-^NES^-178^), the upper band could be *N*-glycosylated FKBP22. The FKBP22 expression constructs contain an additional 6x His tag and enterokinase recognition site at the amino terminus (detailed described in method section), therefore the transfected FKBP22 proteins migrated a little slower than endogenous FKBP22 (arrowhead in Supplemental Fig. S1B). This Western blotting result indicated that the solubility of transfected M48K FKBP22 was almost equal to that of transfected WT FKBP22 in the rER (Fig. 7A). The transfected FKBP22 showed faint bands due to low transfection efficiency of primary fibroblasts (Supplemental Fig. S1B) and the expression vector produced a non-specific antigen against the antibody (black line in Supplemental Fig. S1B). Furthermore, overexpression partially induced approximately 20–30% insoluble material compared to 100% solubility of endogenous WT (Fig. 7A). As the mutant FKBP22 protein formed aggregates more readily than WT FKBP22 when recombinantly expressed in *E. coli*, we explored the hypothesis that the accumulation of aggregated mutant FKBP22 protein in the ER could lead to the activation of an ER stress response. We, therefore, compared the protein level of BiP between vector, WT and M48K transfected FKBP22 null cells. The BiP protein level was normalized by internal control β-tubulin and there was no significant difference in the amount of BiP between the three cells (Fig. 7B).

In conclusion, M48K FKBP22 is properly located in the rER and although its solubility is slightly, but not significantly, reduced compared to WT FKBP22, there is no apparent ER stress activation in the transfected cells.

## Discussion

A novel homozygous c.143 T > A substitution in exon 1 in *FKBP14* replaces Met 48 residue with a Lys residue in FKBP22 protein and causes kEDS^7^. In contrast to the other known mutations in *FKBP14*, the M48K FKBP22 transcript is efficiently translated and does not undergo nonsense-mediated mRNA decay. Due to the lack of patient’s fibroblasts, a biochemical approach with recombinant proteins was firstly used to investigate the effect of the missense variant on FKBP22 functions. Here, we show a basic structural analysis (Fig. 2), enzyme activities (Fig. 3) and protein – protein interactions (Fig. 5) using purified recombinant WT and M48K FKBP22 protein. In addition to *in vitro* studies, we verified the subcellular localization, intracellular solubility and ER stress response of the M48K FKBP22 in the cells using the FKBP22 null cells (Figs. 6,7). We identify type IV collagen as a novel substrate of FKBP22 (Fig. 3). The lens is one of the most enriched tissues for type IV collagen^26,27^, and 60% of patients with *FKBP14*-kEDS suffer from refraction anomalies^7^. On the other hand, muscular impairment is identified in almost 100% of patients^7^. Such tissue specific differences could be attributed to the different binding affinities of FKBP22 to individual collagens since tissues differ in their collagen makeup. Myopathic EDS is caused by heterozygous or biallelic mutations in *COL12A1*, encoding type XII collagen^1,28^. As type IV collagen becomes a novel substrate of FKBP22, we suspect type XII collagen could be another candidate for an interaction with FKBP22 that requires further investigation. Our results demonstrate that the M48K mutation impairs but does not abolish FKBP22 functions. Furthermore, we show that mutant FKBP22 is properly localized in the rER maintaining a similar solubility as WT FKBP22 without ER stress (Figs. 6,7). While there are small aggregates in the final purified product of M48K FKBP22 (Fig. 2), purified mutant protein was stable and did not form further aggregates. The small structural change could induce unfavorable interactions with endogenous *E. Coli* proteins and show a tendency to form aggregates during the purification of the mutant protein. The homology model of M48K FKBP22 suggests that this change is likely to be associated with a modified microenvironment of the FKBP domain and/or surface due to the missense mutation (Fig. 1). It has been proposed that the size and/or shape of the FKBP domain confers substrate recognition and enzyme activity^10,19,29^. Indeed, six different rER resident FKBPs individually show different levels of activity and preferences of post-translational modification of proline residues or the amino acid preceding the proline residue^17^. Additionally, methionine 48 is close to the active site of the FKBP domain. Collectively, we attribute to a small conformational change to the catalytic pocket as this weakened PPIase activity in this mutation. Interestingly, this mutation showed both negative and positive effects of binding to type III/IV/VI collagen and type X collagen, respectively (Fig. 5). The M48K mutation increases the binding of FKBP22 to type X collagen and this K_D_ value is close to the K_D_ values between Hsp47 and collagens^18^. Thus, this change could have M48K FKBP22 compete with Hsp47 during type X collagen biosynthesis and/or secretion. Conversely, the attenuated binding capability to type III/IV/VI collagen could affect the quality control of these collagens in the rER. Unfortunately, it is not yet known how FKBP22 recognizes specific triple helical collagen sequences. Since previous studies showed the disruption of the EF-hands motives did neither change the PPIase activity nor the amount of refolded type III collagen at the end of measurements^18^, the FKBP domain is responsible for the binding to the triple helix of collagen. However, there is no information of the binding orientation between FKBP domain and collagen triple helices, therefore either the active site showing PPIase activity or the opposite side of that site should be involved in collagen binding. Taken together, we propose that the M48K mutation affects not only the microenvironment of the catalytic pocket in the FKBP domain but also the overall structure of the FKBP domain. Further studies are required to determine the precise binding mechanism, which may subsequently explain the enhanced interaction between type X collagen and M48K FKBP22 protein.

In conclusion, the M48K mutation does not affect the expression of FKBP22 in the rER but leads to an FKBP22 with weakened functions. Here we show that a partial loss of function leads to a similar phenotype as the complete loss of FKBP22 even though M48K FKBP22 could still have fully functional EF hand motifs, the function of which is mostly unknown except for binding calcium^30,31^. The molecular findings are in keeping with the clinical findings of the homozygous M48K patient which were similar to these of FKBP22 null patients^7^. In summary, our study provides direct evidence for the effects of the M48K mutation in FKBP22, however additional studies are required to explore the disease mechanism of *FKBP14*-kEDS.

## Experimental Procedures

### Homology model

The structure model of M48K mutant was built by Discovery Studio 4.0 using WT FKBP22 structure (PDB: 4MSP). WT and created mutant structures were applied to surface analyses in Discovery Studio 4.0.

### Cloning, expression, and purification of recombinant WT and mutant human FKBP22

Human M48K FKBP22 mutant was generated using the QuikChange Lightning Site-Directed Mutagenesis Kit (Agilent Technologies). Human WT FKBP22, which was constructed as described previously^18^, was used as a template and the oligonucleotides were designed using the QuikChange Primer Design program provided by Agilent Technologies (http://www.genomics.agilent.com/primerDesignProgram.jsp). 5′-cttcatagtggaccaacttcaaatcccctcctttgg-3′ and 5′-ccaaaggaggggatttgaagttggtccactatgaag-3′ (mutated nucleotides are underlined) were used as the sense and antisense primers, respectively. The DNA sequence of mutant FKBP22 was verified by DNA sequencing (sequetech). The expression vectors were transformed into *Escherichia coli* BL21 (DE3) and grown at 37 °C to an optical density of 0.6 at 600 nm, and expression was induced with 1 mM isopropyl β-D-1-thiogalactopyranoside. After incubation at 20 °C overnight, the cells were harvested by centrifugation and resuspended in Tris base B-PER (Thermo Scientific) containing 1 mM CaCl_2_. Soluble material was removed by centrifugation, and insoluble material was resuspended in column binding/equilibration buffer (20 mM HEPES buffer, pH 7.5, containing 1.0 M NaCl, 20 mM imidazole, 1 mM CaCl_2_ and 2 M urea). After centrifugation, the supernatant was passed through a 0.22-μm filter and loaded onto a Co^2+^-chelating column. After washing with the same buffer (minimum 5 column volumes), mutant FKBP22 was eluted with elution buffer (20 mM HEPES buffer, pH 7.5, containing 1.0 M NaCl, 500 mM imidazole, and 1 mM CaCl_2_). The fractions containing mutant FKBP22 were dialyzed against enterokinase cleavage buffer (50 mM Tris/HCl buffer, pH 8.0, containing 1 mM CaCl_2_ and 0.1% Tween 20). Enterokinase (1 unit/ml reaction volume) (Invitrogen) was used to cleave the His tag at 4 °C overnight and the sample was then dialyzed against column binding buffer. The protein solution, containing mutant FKBP22, was treated with 1 μl/ml diisopropyl fluorophosphate to inactivate residual enterokinase and proteases derived from *E. coli* by gentle stirring for 4 h on ice. This solution was then applied to a Co^2+^-chelating column to remove the cleaved His tag fragment. The flow-through fraction containing mutant FKBP22 was dialyzed against 20 mM Tris/HCl buffer, pH 7.5, containing 1.0 M NaCl and 1 mM CaCl_2_ and loaded onto a Phenyl column (GE Healthcare) which was then washed with the same buffer (minimum 5 column volumes). Contaminating proteins were removed by washing the column with 20 mM Tris/HCl buffer, pH 7.5, containing 1 mM CaCl_2_ and mutant FKBP22 was then eluted with water. The purified mutant FKBP22 was then dialyzed against 20 mM Tris/HCl buffer, pH 7.5, containing 1 mM CaCl_2_ and used for further experiments.

### Circular dichroism measurements

Circular dichroism spectra were recorded on an Aviv 202 spectropolarimeter (Aviv, Lakewood, NJ) using a Peltier thermostatted cell holder and a 1-mm path length cell (Starna Cells, Atascadero, CA). Protein concentrations were determined by amino acid analysis. The spectra represent the average of at least 3 scans recorded at a wavelength resolution of 0.1 nm. The proteins were measured in 1 mM Tris buffer containing 0.05 mM CaCl_2_, pH 7.5, at 4 °C.

### Refolding of full-length type III collagen measured by circular dichroism

Refolding was monitored by circular dichroism measurements at 220 nm. Isolation and purification of bovine type III collagen was as described previously^18^. Collagens were denatured for 5 min at 45 °C and then added into precooled reaction buffer (50 mM Tris/HCl, pH 7.5, containing 0.2 M NaCl and 1 mM CaCl_2_) at 10 °C in the absence and the presence of either wild-type or mutant FKBP22. Refolding was monitored for 4500 s at 10 °C. Protein concentrations were 0.2 μM for type III collagen and 2 μM for wild-type and mutant FKBP22. All curves are the average of at least three independent measurements.

### Stopped-flow enzyme assays

Measurements of the catalytic efficiency (*k*_cat_/*K*_m_) for the isomerization reaction were performed as previously described^17,18^. Kinetic measurements were made at 5 °C to minimize the non-enzymatic isomerization reaction and were carried out in 35 mM HEPES buffer, pH 7.8, containing 1 mM CaCl_2_. The Suc-Ala-Gly-3Hyp-Phe-pNa peptide (where pNA is *p*-nitroanilide) was purchased from KareBay Biochem Inc. (Monmouth Junction, NJ). Stock solutions of substrates were prepared in DMSO and the final concentration of peptide was 225 µM. Chymotrypsin and DMSO final concentrations were 75 µM and 0.25%, respectively. Absorbance change was monitored for pNa at 390 nm with a HiTech stopped-flow spectrophotometer (TgK Scientific Limited, Bradford-on-Avon, UK). The assay was started by mixing chymotrypsin and the substrate peptide. Data points were collected using Kinetic Studio version 4.06 software (TgK Scientific Limited, Bradford-on-Avon, UK) and progression curves were analyzed by fitting to a second-order exponential-decay function using ORIGIN Pro ver. 9.1 (OriginLab Corp., Northampton, MA). The slower rate of the decay was used as the cis/trans isomerization reaction. Values for *k*_*cat*_*/K*_*m*_ were calculated according to *k*_cat_/*K*_m_ = (*k*_obs_–*k*_u_)/[E], where *k*_*u*_ is rate constant for the unanalyzed isomerization reaction and *k*_*obs*_ is the rate constant for the catalyzed reaction in the presence of enzymes at a given concentration of [E]. *k* values were calculated using ORIGIN assuming that the reaction consists of two first-order reactions, the fast phase due to the cleavage of the initial *trans* form of the Gly-3Hyp peptide bond, and the slow phase due to the catalyzed or uncatalyzed cis to trans isomerization followed by the fast peptide bond cleavage. To detect the catalysis, a maximum final concentration of enzyme of 0.5 µM was used in this assay.

### Surface plasmon resonance (SPR) analysis

SPR experiments were carried out using a BIAcore X instrument (GE Healthcare). Purified type III, type IV, type VI, type X and type XI collagen were immobilized on CM5 sensor chips by amide coupling. Isolation and purification of bovine type III collagen were described above. Mouse type IV collagen and human recombinant type X collagen were purified as described previously by using HR9 and 293 cells, respectively^32,33^. Bovine type XI collagen was purified by the method described previously^34^. Human type VI collagen was provided by Takako Sasaki, Department of Biochemistry II, Faculty of Medicine, Oita University, Oita, Japan. The approximate coupled protein concentrations were 3.0 ng/mm^2^ (3000 response units (RU)) of type III collagen, 4.5 ng/mm^2^ (4500 RU) of type IV collagen, 4.5 ng/mm^2^ (4500 RU) of type VI collagen, 3.0 ng/mm^2^ (4500 RU) of type X collagen and 0.95 ng/mm^2^ (950 RU) of type XI collagen. The experiments were performed at 20 °C in HBS-P (10 mM Hepes, pH 7.4, containing 150 mM NaCl and 0.005% Surfactant P20) containing 1 mM CaCl_2_ and using a flow rate of 10 µL/min. All curves are the average of at least three replicates, and three independent measurements were performed. For analysis of binding affinity, the curves were fitted with the steady-state affinity model and the Langmuir-binding model for type IV collagen – WT FKBP22 and type X collagen – M48K FKBP22, respectively (BIAevaluation software; GE Healthcare).

### Construction of wild type and mutant FKBP22 for mammalian expression

RNA was isolated from HEK293 cells and first strand cDNA was then synthesized from this RNA according to standard protocols using the Superscript III system (Invitrogen). The cDNA coding for WT FKBP22 lacking its natural signal peptide was then amplified using the following primers, each containing a *Hind III* restriction site: Forward 5′-AAGCTTGCTTTGATCCCTGAACCAGAAGTG-3′ and Reverse 5′-AAGCTTCTATAACTCATCGTGTTTATATGTAAATTCTC-3′. The PCR product was digested with *Hind III* and cloned into the *Hind III* site of the expression vector pCEP4-BM40-hisEK that contains the sequence for the BM40 signal peptide followed by a 6x His tag, enterokinase recognition site and also confers Hygromycin resistance^35^. Insert orientation and correct sequence were confirmed by sequencing. This construct was then used as a template for the generation of Mutant FKBP22 in the same manner as for the bacterial expression construct and successful mutagenesis was verified by sequencing.

### Cell culture and transfection

Primary fibroblast cultures were established from skin biopsies of healthy and affected individuals by explant culture. Human tissues were obtained at the University Children’s Hospital (Zurich, Switzerland) for diagnostic purposes. Ethics approval was granted by the Swiss Federal Ethic committee Swiss Ethics (KEK Ref.-Nr.342 2014–0300 and Nr. 2019–00811) in the presence of a signed informed consent of the patients. This study has been conducted in accordance with the Helsinki declaration and its following modifications. Fibroblasts grown from patients skin biopsies were routinely maintained at 37 °C in a 5% CO_2_ atmosphere and grown in standard DMEM (Dulbecco modified essential medium; Gibco, 31966) culture medium supplemented with 10% fetal calf serum, 100 units/mL of penicillin, 100 µg/mL of streptomycin, 0.25 µg/mL of amphotericin B (Gibco) and 5 mM Hepes in presence of ascorbic acid phosphate (100 μg/mL; Wako Chemicals). A plasmid of pCEP-BM40HIS vector, WT or M48K FKBP22 expression constructs were transfected into FKBP22 null human fibroblasts using TransIT-X2 Dynamic Delivery System (Mirus) according to manufacturer’s instructions. Experiments were performed 24 hours after transfection.

### Antibodies

Rabbit polyclonal antibody against BiP (abcom; ab21685), rabbit polyclonal antibody against β-Tubulin III (Sigma-Aldrich; T2200), Amersham ECL rabbit IgG, HRP-linked whole Ab from donkey (GE Healthcare Life Sciences; NA934) were used for Western blotting. Mouse monoclonal antibody against KDEL (10C3) (Enzo Life Sciences; ADI-SPA-827) and Alexa Fluor 488-conjugated anti-mouse (Thermo Fisher Scientific; A-11017) and Alexa Fluor 568-conjugated anti-rabbit IgG (Thermo Fisher Scientific; A-21069) were used for immunofluorescent staining. Rabbit polyclonal antibody against FKBP22/*FKBP14* (15884-1-AP; proteintech) was used for both Western blotting and immunofluorescent staining.

### Immunofluorescent staining

Immunofluorescent staining was performed using Nunc Lab-Tek Chamber Slide System (Thermo Fisher Scientific). The cells were fixed by 4% PFA and permeabilized with PBS containing 0.1% Triton X-100 after washing with PBS. The cells were incubated with specific antibodies at room temperature for 2 h after blocking with PBS containing 10% glycerol, 2% goat serum and 0.02% Trinton X-100. The dilution ratio of primary antibodies was 1:200 for both FKBP22 and KDEL antibody. After incubation with antibodies and washing in PBS solution, fluorophore-conjugated secondary antibodies were added to the cells at a 1:1,000 dilution in the dark for 30 min. After washing in PBS solution, a coverslip was placed with the aqueous mounting medium Fluoroshield with DAPI (Sigma-Aldrich) and stored in the dark overnight at room temperature. Immunofluorescence was observed with a Zeiss Axio Imager M1 microscope, and micrographs were recorded digitally using the AxioVision software (AxioVs40x64 V 4.9.1.0).

### Western blot analysis

Cell lysates were extracted using M-PER (Thermo Fisher Scientific) containing Halt™ Protease Inhibitor Cocktail, EDTA-Free (Thermo Fisher Scientific) at 4 °C according to manufacturer’s instructions. After centrifugation, soluble proteins in the supernatant and insoluble proteins in pellet fraction were mixed with SDS sample buffer with reducing agents. These protein solutions were separated on NuPAGE 12% Bis-Tris gel (Thermo Fisher Scientific) or Novex WedgeWell 12% Tris-Glycine gel (Thermo Fisher Scientific) then transferred to PVDF membranes (Bio-Rad) and Western blots were performed using antibodies listed in above. Blots were developed with HRP enhanced SuperSignal West Pico Chemiluminescent Substrate (Thermo Fisher Scientific) and detected by ChemiDoc MP imaging system (BioRad) using the software Image Lab version 4.0.1 (BioRad). The intensities of protein signals were measured by Image J.

### Statistical analyses

For comparisons between two groups, we performed one-way ANOVA using ORIGIN Pro ver. 9.1 (OriginLab Corp., Northampton, MA) to determine whether differences between groups are significant. A p-value of less than 0.05 was considered statistically significant.

## Supplementary information

Supplemental Figure.
