# Supplementary material for: The novel missense mutation Met48Lys in FKBP22 changes its structure and functions
Source: Sci Rep. 2020 Jan 16;10:497. doi: 10.1038/s41598-019-57374-y (PMC6965642; doi:10.1038/s41598-019-57374-y)
Supplement: Supplementary file 1 — Supplemental Figure. [file 41598_2019_57374_MOESM1_ESM.pdf]

Supplementary Information

The novel missense mutation Met48Lys in FKBP22 changes its structure and functions

Yoshihiro Ishikawa[1,2,3,*], Nobuyo Mizuno[2,4], Paul Holden[2], Pei Jin Lim[5], Douglas B. Gould[3,6], Marianne Rohrbach[5], Cecilia Giunta[5] and Hans Peter Bächinger[1]

1: Department of Biochemistry and Molecular Biology, Oregon Health & Science University, Portland OR USA
2: Shriners Hospital for Children, Research Department, Portland OR USA
3: Department of Ophthalmology, University of California, San Francisco, School of Medicine, CA USA
4: Vaccine and Gene Therapy Institute, Oregon Health and Science University, Portland, Oregon, USA
5: Connective Tissue Unit, Division of Metabolism and Children's Research Centre, University Children's Hospital, Zürich, Switzerland
6: Department of Anatomy, University of California, San Francisco, School of Medicine, CA USA

* Corresponding author:
Yoshihiro Ishikawa, Ph.D.,
Department of Ophthalmology, University of California, San Francisco, School of Medicine, CA USA;
E-mail: yoshi.molecularensemble@gmail.com
Tel. (503)866-5940

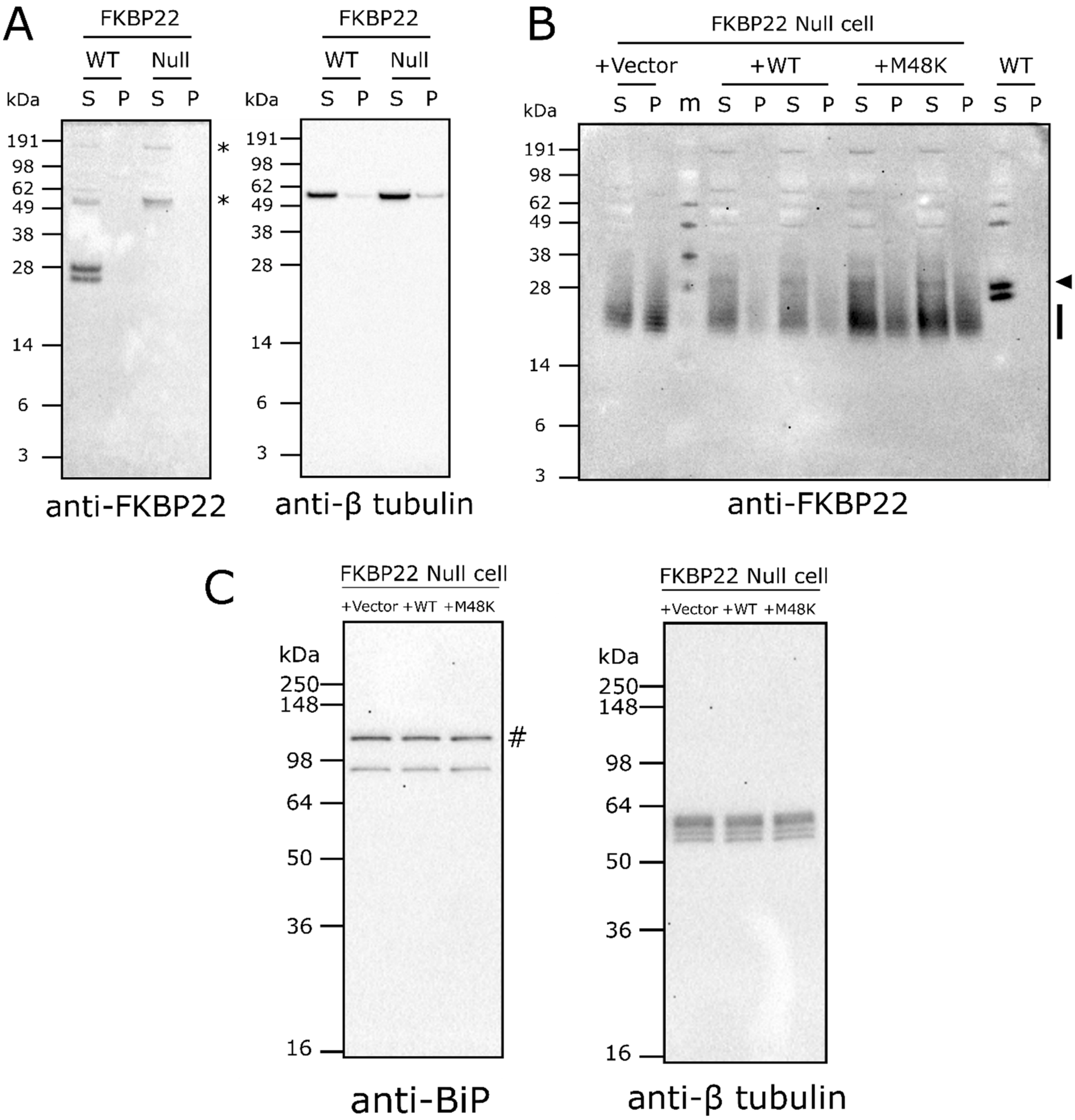

Supplemental Figure S1. Western blotting analysis of intracellular solubility of human WT and M48K FKBP22 and protein level of BiP in transfected FKBP22 Null human primary fibroblasts.
(A) Western blotting was performed to evaluate the detection of FKBP22 in WT and FKBP22 Null human primary fibroblasts. S and P indicate the supernatant and pellet fraction after centrifugation. Asterisks indicate the non-specific band detected by anti-FKBP22. Proteins were separated on a NuPAGE 12% Bis-Tris gel (Thermo Fisher Scientific) and blotted onto PVDF membrane (BioRad). (B) Western blotting was performed to evaluate the solubility of transfected human WT and M48K FKBP22 in FKBP22 Null human primary fibroblasts. Two independently prepared samples were loaded for transfected WT and M48K FKBP22. S and P indicate the supernatant and pellet fraction after centrifugation. Marker lane is shown as “m”. Arrowheads point to the location of transfected FKBP22. Black line indicates the non-specific bands produced by expression plasmids. Proteins were separated on a NuPAGE 12% Bis-Tris gel

(Thermo Fisher Scientific) and blotted onto PVDF membrane (BioRad). (C) Western blotting was performed to see effects on protein level of ER stress marker BiP in FKBP22 Null human primary fibroblasts after transfection of vector, FKBP22 WT and M48K construct. Pound sign in anti-BiP is the non-specific band verified at vender website (https://www.abcam.com/grp78-bip-antibody-ab21685.html). Proteins were separated on a Novex WedgeWell 12 % Tris-Glycine gel (Thermo Fisher Scientific) and blotted onto PVDF membrane (BioRad). All images of Western blotting in this figure were scanned by ChemiDoc MP imaging system (BioRad) using the software Image Lab version 4.0.1 (BioRad) and then the original scanned image was used to create individual figures.
